# The Effects of Host Diversity on Vector-Borne Disease: The Conditions under Which Diversity Will Amplify or Dilute the Disease Risk

**DOI:** 10.1371/journal.pone.0080279

**Published:** 2013-11-26

**Authors:** Ezer Miller, Amit Huppert

**Affiliations:** 1 The Hebrew University of Jerusalem, The Faculty of Medicine, Microbiology and Molecular Genetics Department, Jerusalem, Israel; 2 Gertner Institute for Epidemiology and Health Policy Research, Biostatistics Unit, Ramat Gan, Israel; Instituto de Higiene e Medicina Tropical, Portugal

## Abstract

Multihost vector-borne infectious diseases form a significant fraction of the global infectious disease burden. In this study we explore the relationship between host diversity, vector behavior, and disease risk. To this end, we have developed a new dynamic model which includes two distinct host species and one vector species with variable preferences. With the aid of the model we were able to compute the basic reproductive rate, *R*_0_, a well-established measure of disease risk that serves as a threshold parameter for disease outbreak. The model analysis reveals that the system has two different qualitative behaviors: (i) the well-known dilution effect, where the maximal *R_0_* is obtained in a community which consists a single host (ii) a new amplification effect, denoted by us as diversity amplification, where the maximal *R_0_* is attained in a community which consists both hosts. The model analysis extends on previous results by underlining the mechanism of both, diversity amplification and the dilution, and specifies the exact conditions for their occurrence. We have found that diversity amplification occurs where the vector prefers the host with the highest transmission ability, and dilution is obtained when the vector does not show any preference, or it prefers to bite the host with the lower transmission ability. The mechanisms of dilution and diversity amplification are able to account for the different and contradictory patterns often observed in nature (i.e., in some cases disease risk is increased while in other is decreased when the diversity is increased). Implication of the diversity amplification mechanism also challenges current premises about the interaction between biodiversity, climate change, and disease risk and calls for retrospective thinking in planning intervention policies aimed at protecting the preferred host species.

## Introduction

Vector-borne infectious diseases form a significant fraction of the global infectious disease burden [1]–[3]. For instance, the World Health Organization estimates the collective human death due to vector borne diseases to be more than 1.5 million per annum, particularly of children in the developing world [3]. Moreover, recent studies indicate that the disease burden of several major vector-borne diseases is on the rise [3]. Most vector-borne diseases can infect multiple host species [4], [5]. Important examples include malaria, Leishmaniasis, yellow fever, Lyme disease and the West Nile Virus [4]–[7].

Recent studies indicate that when host diversity i.e., either species richness (no. of species) or their evenness increases, the disease risk can either decrease or increase in what is known as the dilution effect [6], [8]–[19]. The common mechanism given in the literature behind the dilution effect is rather simple; if an increase in host diversity decreases or increases the probability that the vector will come across a high competent host, i.e., a host with a higher ability to transmit the respective pathogen [17], [20], a dilution or amplification will occur, respectively. Therefore, the greater the abundance of inferior/superior competent host species, the lower/higher the probability of disease transmission and the stronger is the dilution effect [6], [8], [14]–[17]. Using the above logic, it is clear that the maximal disease risk is always obtained when the community is composed entirely of the most competent host species.

Yet, several studies have questioned the universality of the dilution effect by providing evidence that maximum disease risk is obtained when the community consists of several host species [20]–[23]. Furthermore, a recent review by Randolph and Dobson even disputes the interpretation of the results obtained previously by studies which support the dilution mechanism [24].

Mathematical models are a vital tool which can be used to resolve complex biological phenomena such as multihost transmission. Yet, theoretical studies on multihost diseases are sparse; Keesing et al. 2006 have used a single species susceptible - infected -removed (SIR) type model [25] to explore how modifications of certain parameters which mimic the effect of biodiversity, affect the disease dynamics [11]. Dobson [26] has developed a general framework to study the relationship between host diversity and disease prevalence. The Dobson model explicitly includes multiple host species. Both models are very general, and as such, are not focused on vector borne diseases and do not incorporate the vector preference, although many species of vectors present significant preference toward certain host species and even for certain individuals within the same host population [27]–[30]. A vector preference induces heterogeneous biting among the host individuals which has been found to significantly increase the disease risk in both, field studies and models [20], [27], [31]–[33]. Moreover, a multihost vector-borne disease model which was designed to reproduce the variations in the intensity of the West Nile Virus in several sites in Connecticut indicated that the vector's (mosquitoes) feeding preference was the most significant factor that influenced both, the peak timing and the intensity of the disease outbreaks [20].

Here we present and analyze a novel dynamic model which explores how the combination of host diversity, host transmission ability, and vector preference, affect the disease risk. The modeling framework is based on that of Ross [34] which was extended to include two distinct host species as in Simpson et al. 2012 [20], one vector species with variable preferences, and unlike previous studies, a critical separation between density and diversity parameters (richness and evenness) [11], [13], [19]. The model analysis reveals a new mechanism for disease amplification for which the maximum in disease risk is obtained when both host species are present in the community. We therefore denote it as *diversity amplification*. Our model expands on the previous understanding about the relationship between host diversity and disease risk by formulating the exact conditions under which diversity amplification, or dilution, would occur. More specifically, we have found that diversity amplification occurs when the preferred host species is also the one with the highest transmission ability, while dilution occurs when the vector prefers to bite the host with the lower transmission ability, or it does not have preference at all. The mechanisms of diversity amplification and dilution are able to account for the different and contradictory patterns often observed in nature (i.e., disease outbreaks are sometime enhanced while in other cases are suppressed when the community diversity is increased). Furthermore, the diversity amplification mechanism also calls for retrospective thinking in planning future intervention policies in order to mitigate the burden of multihost vector borne diseases.

## Methods

### Model description

We model a vector-borne disease with two species of hosts and one species of vector by using a dynamic compartmentalized model. Each host population is partitioned into susceptible, infected and removed compartments, while vector individuals can only be susceptible or infected. All host populations are assumed constant, i.e., in the model timescale, birth and death rates of the hosts are negligible compare to these rates in the vector population and the disease recovery rate. The vector population, however, unlike the hosts, is assumed at dynamic equilibrium (i.e., individuals are born and die while maintaining a fixed number, *V*). In our model, newborn vectors are always susceptible (there is no vertical transmission), while those which are infected (as a result of feeding on an infected host), do not recover but stay infected till they die. We assume that both, susceptible and infected vectors are born (at rate *r*) and die (at rate *d*) at the same rates, i.e., the disease parasites do not affect vector longevity and fertility (equations 1.e, 1f). These assumptions are therefore a SIR (Susceptible-Infected-Recovered) generalization of the Ross model [33] which includes multiple hosts [20], [34]. The following system of six equations (equations 1a-f) represents the dynamics of the model compartments (Susceptible and Infected individuals of each population): 
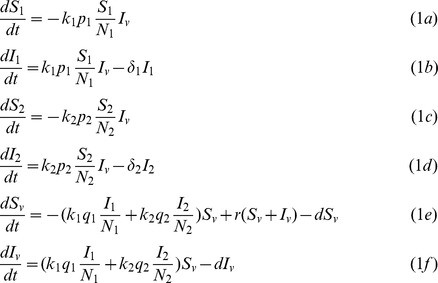



In equation 1, *S_i_* and *I_i_* are the susceptible and infected individual number of host species *i* (1≤i≤2, equations 1a-d), and *I_v_* and *S_v_* are the infected and susceptible number of the vectors (equations 1e, f). The force of infection of each host (i.e., the number of infections per unit time) is given by *k_i_p_i_S_i_I_v_/N_i_*, (1≤i≤*2*, equations 1a-d) where *k_i_*, and *p_i_*, are the bite rate and the transmission efficiency between the vector and host *i*, respectively, and *N_i_* is the host *i* fixed population size. Each host species is recovered at rate δ*_i_* (equations 1b-d). The removed compartments of the various hosts *R_i_,* can be calculated by using the *S_i_* and *I_i_* equations (equations 1a-d) since for host *i* with fixed population size *N_i_*, we have *R _i_* =  *N_i_* – *I_i_* – *S_i_*. The force of infections of the vectors is the sum of the infections caused by the two host species in the community (equations 1e, f). A list of all the model (equation 1) parameters and their meanings can be found in table 1.

**Table 1:** The meaning of the model parameters.

Parameter	Meaning
***I_i_***	Number of Infected host species i
***S_i_***	Number of Susceptible host species i.
***I_v_***	Number of Infected vectors.
***S_v_***	Number of susceptible vectors.
***k_i_***	Bite rate, i.e., the number of bites per unit time between the vector and host species i.
***N_i_***	Host i population size.
***V***	Vector population size.
***N***	The total density of hosts in the community. i.e., *N* = *N_1_*+*N_2_*
***p_i_***	The efficiency that an infected vector would infect a susceptible individual of host species i during one feeding event.
***q_i_***	The efficiency that an infected individual of host species i would infect a susceptible vector during one feeding event.
***δ_i_***	Recovery rate of host i, i.e., 1/δ_i_ is the disease duration.
***r***	The vector intrinsic rate of increase.
***d***	Vector death rate.
***k***	The total bite rate of the vector with the entire community, i.e., *k* = *k_1_*+*k_2_*
***g_i_***	The transmission ability of host species species i. *g_i_* = *p_i_q*_i_/*δ_i_*
**γ**	The transmission ratio γ = g_2_/g_1_.
**α**	The vector preference. α = (*k*_2_/*k*_1_)/(*N*_2_/*N*_1_)
***x***	The proportion of species 1 in the community, i.e., *N_1_* = *xN* and *N_2_* = (1–*x*)*N*.

### Model analysis

In this study we use *R*_0_, the basic reproductive rate, as a disease risk measure.

The basic reproductive rate, *R*_0_, has played a crucial role in the epidemiological theory of infectious diseases because it forms a measure for disease onset intensity, and establishes a threshold criterion for their eruption [25], [33]. Generally speaking, *R*_0_ is a threshold parameter which determines the stability of the Disease Free Equilibrium (DFE) point, i.e., an equilibrium for which *I_i_* = 0 and *I_v_* = 0 (1≤i≤*2*). If *R_0_*>1 the DFE is unstable and the disease can invade the community and if *R_0_*<1 the DFE is locally asymptotically stable and the disease will never erupt [25], [35]. In our model, this mathematical definition of *R*_0_ can be biologically interpreted as the number of secondary infections caused by an individual infected host during the disease duration and the vector longevity (equation 2). The basic reproduction rate, *R_0_*, was calculated by using the next generation operator technique [35]. Details on this calculation can be found in Appendix S1.
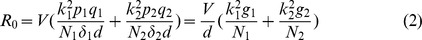



The transmission ability, *g_i_* = *p_i_q_i_/δ_i_* (table 1), is the efficiency that a vector which bites an infected host species *i*, would infect a susceptible host of the same host species ( = *p*_i_ *q_i_*) during its disease duration (1/δ*_i_*). The higher the *g_i_*, the better the host species *i* is as a disease transmitter. Note that if *g_i_* = 0, the host can neither infect nor be infected with the respective disease i.e., it is a dead end host.

Similar expressions for *R_0_* of dynamic models of vector-borne diseases were obtained for both, multi-host community [36] and a metapopulation version of a single-host model for which the *k_i_*'s represent the proportion of vector individuals which bite hosts in patch *i* [31].

Our goal in this study is to explore how *R*_0_ depends on species diversity. To this end, we denoted in *N* the total host density within the community, and in *x* the proportion of species 1 (0≤*x*≤1). The two host species densities, *N_1_* and *N_2_*, are therefore given by *N_1_* = *xN* and *N*_2_ *= *(*1*–*x*)*N* (so *N* = *N_1_*+*N*_2_). Since our community consists only two species, species richness and evenness are determined by *x*: if *x* = 0 or 1, the community consists of a single species and if 0<*x*<1 the community consists of two species with variable evenness which are determined by the particular value of *x*. The evenness would be maximal for *x* = 0.5, and minimal when *x* = 1 or 0. To simplify the analysis we introduce two dimensionless parameters which play an important role in determining the behavior of *R*_0_ as a function of host diversity (richness and evenness). The first is the vector preference α (table 1):
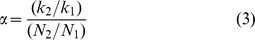



i.e., the preference is the deviation between the bite rates (*k*_i_) ratio and the host densities/population sizes ratio. A somewhat similar parameter also serves as an index in field studies to measure vector preferences for various host species [28]. According to equation 3, the vector shows no preference to any host for α = 1. When α>1 or 0<α<1 the vector prefers to bite host species 2 or 1, respectively.

The second parameter is the transmission ratio, γ (table 1):
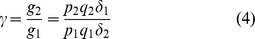



i.e., γ measures the relative disease transmission of host species 2 compared to that of host species 1. Similar to the definition of α, when γ = 1, the two hosts have equal transmission ability and when γ>1 or 0<γ<1, the better disease transmitter is host species 2 or 1, respectively. The host competence in this model is therefore determined by two parameters. The transmission ratio, γ, which depends on the disease internal epidemiology of the two hosts (such as the disease duration, the transmission efficiency of an infectious bite, etc.), and the vector preference, α, which depends on the vector behavior (e.g., its affinity for certain visual or chemical cues, etc.).

One of the crucial factors that determine the disease dynamics and the way *R*_0_ depends on species diversity is the way the vector divides its bites between the two host species as a function of its preference and the host species composition. In the model the vector is assumed to have a frequency-dependent (density-independent) biting rate i.e., the total number of bites an individual vector has per unit time, *k*, is independent of host densities (it is assumed that the density of the hosts is high enough and does not limit the vector bite rate, which attains its maximal value) [25], [34], [36]. The expressions of the vector bite rates with each host species as a function of its preference, α, and the proportion of species 1, *x*, are given in equation 5. For the derivation of these expressions see Appendix S1. 
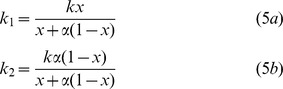



(*k_i_* are the bites rates, i.e., the number of bites an individual vector has with host species *i* per unit time). Note that *k* = *k*_1_+*k*_2_, i.e., the total bite rate is independent on *N*, *x*, and *α*.

With the aid of equations 2–5, we obtain *R*_0_ as a function of *x*: 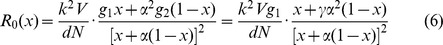



The main goal of this paper is to explain the mechanisms of the dilution and the amplification in disease risk by exploring how *R*_0_ changes both qualitatively and quantitatively as a function of the host composition in the community, *x* (see table 1 for the definitions of all *R_0_* parameters). A complete analysis of *R*_0_(*x*) in equation 6 can be found in Appendix S1.

## Results

Without the loss of generality, we assume from now on that α≥1, (i.e., species 2 is the preferred host by the vector). The analysis reveals that *R_0_*(*x*) can have two main different qualitative behaviors, depending on the relative magnitude of both, the transmission ratio (γ) and the vector preference (α), as we elaborate below (see Appendix S1 for the complete analysis). It is easy to verify (equation 6) that *R_0_* can also be independent of species proportion(*x*). This degenerated case occurs when the vector has no preference and the transmission abilities of both host species are equal (i.e., γ  =  α  =  1, equation 6). In such a case the model collapses to the classic single host species model [33], i.e., *R_0_*(*x*)  =  *k^2^Vg_1_/Nd*  =  *k^2^Vg_2_/Nd*  =  constant.

### Case I: Dilution - *R_0_* is monotonic with species proportion (*x*)

**Figure 1:**
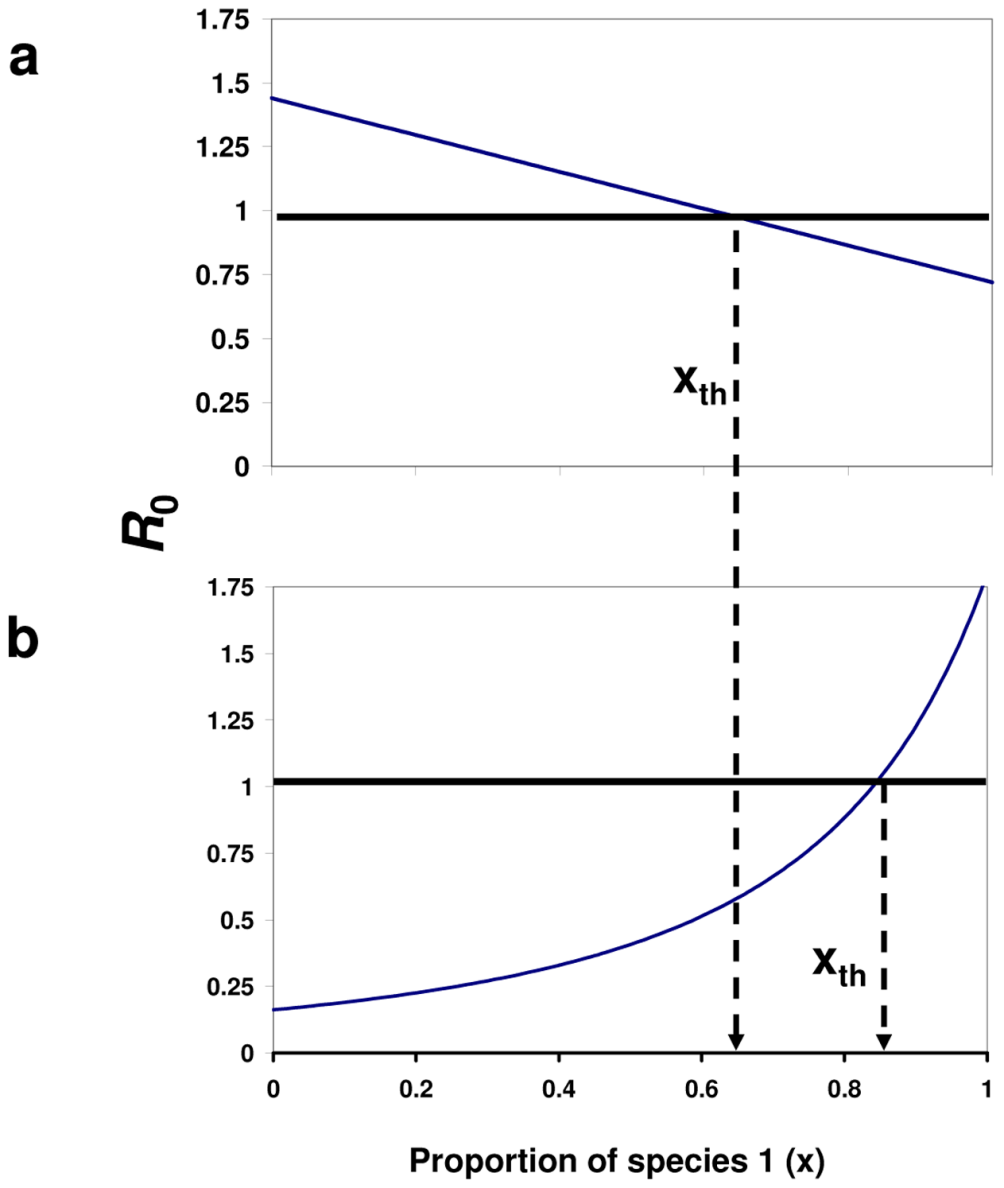
Dilution - *R*_0_(*x*) is monotonic as a function of host diversity. The figure depicts *R_0_* as a function of *x*, the proportion of host species 1 in the community. The behavior of *R*_0_(*x*) depends on the values of the vector preference, α, and the transmission ratio, γ. Panels a and b exemplify two cases of monotonic dependency of *R_0_*(*x*): (a) α = 1 and γ = 2, i.e., there is no vector preference and species 2 is a better transmitter. In this case *R_0_* linearly decreases with an increase in the proportion of species 1, *x* (the inferior transmitter). In this simulation the vector and the total host densities are *V* = 100 and *N* = 2500, respectively. (b) α = 3 and γ = 0.09<1/α^2^, i.e., There is preference towards species 2 and species 1 is the better transmitter. *R_0_* increases with the proportion of species 1 in a nonlinear way. In this simulation the vectors and the total host densities are *V* = 100 and *N* = 1000, respectively. Disease eruption is possible only if *R_0_*>1, i.e., the proportion of species 2 should be above *x_th_* = 0.37 in figure 1a and the proportion of species 1 should be above *x_th_* = 0.84 in figure 1b. Additional parameter values of both figures are: *g_1_* = 0.01, *k* = 3, and *d* = 0.05.

The analysis of equation 6 (see Appendix S1) shows that if (sufficient condition):
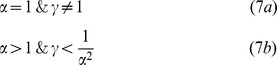



*R_0_* increases monotonically with the proportion of the better transmitter (the species with the higher transmission ability, *g_i_*) host in the community (figure 1). Equation 7a can be related to what previous studies have denoted as a dilution in disease risk; when the vector has no preference (α = 1), the disease risk increases/decreases with the proportion of the species with the higher/lower transmission ability (*g*) [6], [9], [14]–[17]. The condition in equation 7b indicates that the dilution effect can also be obtained when the vector shows preference for the host species which has low enough transmission ability (i.e., the transmission ratio should be below 1/α^2^, equation 7b).

It is easy to verify by examination of equation 6 that when α = 1 (equation 7a), i.e., when the vector shows no preference to any host, *R_0_* is linear with host diversity (*x*) (figure 1a). However, when the condition in equation 7b is met, the dependency of *R_0_* on host diversity (*x*), although monotonic, is not linear. i.e., a change in *R_0_* due to a fixed change in species proportion *x* is not uniform and depends on the particular value of *x* (figure 1b).

When *R_0_*(*x*) is monotonic with species proportion, it obtains its maximal and minimal levels when the community consists of a *single* host species (as predicted by the dilution effect) i.e., the extreme values of *R_0_*(*x*) are always obtained at the edges of the [01] interval (figure 1). An interesting and important case arises near the outbreak threshold *R*_0_ = 1. In such a case the disease can invade the community only when the proportion of the species with the higher transmission ability is above a certain threshold, *x_t_*_h_, 0<*x_th_*<1 (figure 1).

### Case II: Diversity Amplification- *R_0_* is hump-shaped as a function of species proportion (*x*)

**Figure 2:**
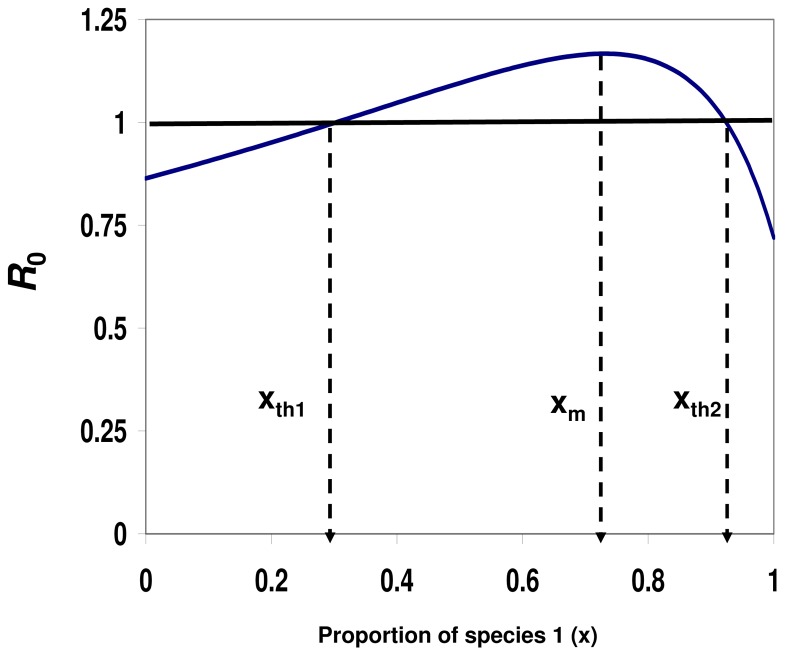
Diversity amplification - *R*_0_(*x*) is hump-shaped as a function of host diversity. The figure depicts *R_0_* as a function of *x*, the proportion of host species 1 in the community. The behavior of *R*_0_(*x*) depends on the values of the vector preference, α, and the transmission ratio, γ. The preference and the transmission ratio in this case are, α = 3.3 and γ = 1.2 (equation 8b), respectively, i.e., species 2 is both, the preferred host and a superior transmitter of the disease. Under such conditions, *R_0_*(*x*) is hump-shaped with a maximum at *x* = *x_m_* =  0.730 (equation 9). Disease eruption is possible only for *R_0_*>1, i.e., when the proportion of species 1, *x*, is in the range *x_th_*_1_ =  0.31 <*x*<0.94 = *x_th_*_2_, or alternatively, when the proportion of the superior host (by both measures; transmission ability and preference), species 2, is between 0.06 and 0.69. Other parameter values are: *V* = 100, *N* = 2500, *g*_1_ = 0.01, *k* = 3, and *d* = 0.05.



The analysis of equation 6 indicates (see Appendix S1 for further details) that when (sufficient conditions):
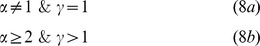



*R_0_* is no longer monotonic with species proportion *x*, but has a maximum value in the open interval (0 1) with a unique maximum point (i.e., it is hump-shaped) at *x* = *x_m_* (see figure 2) where:

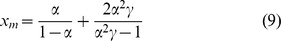



**Figure 3:**
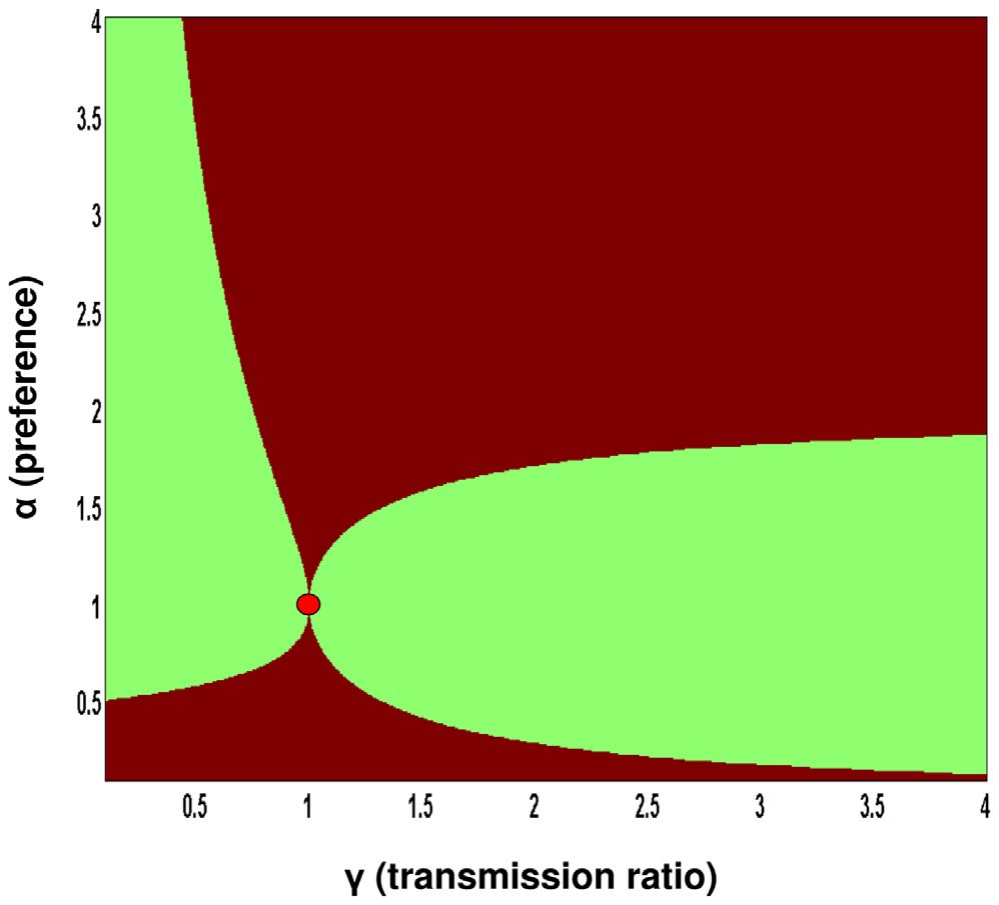
The qualitative behavior of *R*_0_ as a function of the model parameters. The qualitative way by which *R*_0_ depends on the values of the vector preference α, and the host transmission ratio, γ. Brown: *R*_0_ is hump-shaped (diversity amplification), Green: *R*_0_ is monotonic (dilution effect), Red: *R*_0_ is constant (independent on species diversity). Note that *R*_0_ is constant only at a single point where α = γ = 1, a case which is equivalent to a single host model. The red point is therefore disproportionally emphasized. It is apparent that diversity amplification is at least as common as the dilution effect in this parameter range. (i.e., 0.1≤γ≤4, 0.1≤ α ≤4).

As with the monotonic case (case I), an interesting scenario occurs around the outbreak threshold (*R_0_* = 1), when the maximal and minimal values of *R_0_*(*x*) are above and below 1, respectively (figure 2 **).** Under such circumstances, the outbreak is limited to a range of host composition (*x*), given by *x_th1_*<*x*<x_th2_ where *x_th1_* and *x_th2_* are two threshold proportions for which 0<*x_th1_*<1 and 0<*x_th2_*<1 (figure 2). Note that unlike the classic dilution mechanism, the maximum of *R*_0_ in this case is obtained for a specific community composition which contains *both* hosts. We therefore term this mechanism as *diversity amplification*. The condition formulated in equation 8 for the occurrence of diversity amplification (hump-shaped *R_0_*(*x*)) seems to encompass a wide range of vector borne disease systems; it simply requires that the vector should prefer the species with the higher transmission ability (equation 8b). Under this mechanism, therefore, the disease risk as measured by *R*_0_ does not monotonically increase with the proportion of the more competent host (by both measures, transmission ability and vector preference, figure 2, equation 8b). The diversity amplification occurs also when one of the species is a dead end host, i.e., a host for which its transmission ability, *g_i_* = 0. A dead end host therefore serves only as a blood source for the vector and does not participate in the disease transmission cycle. Equation 8b indicates that if the dead end host is the less preferred species (species 1), i.e., *g*_1_ = 0 so γ→∞ (equation 4), and the vector preference α>2, then *R*_0_ will be hump-shaped with a maximum at *x_m_* = 2+α/(1-α) (equation 9). Since the model is general and the conditions of equation 8 are rather broad, we suspect that the diversity amplification mechanism is a common phenomenon in nature. For example, figure 3 demonstrates that diversity amplification (case II) occurs in more than 50% of a symmetrical α- γ space (i.e., when both, α and γ are between 0.1 and 4). Note than in figure 3 the range of the vector preference α includes also cases where α<1. See Appendix S1 for the complete conditions which determine the behavior of *R*_0_(*x*).

### The mechanisms of the dilution and the diversity amplification

The model presented in this paper indicates three types of dependencies (i.e., dilution, diversity amplification, and no affect) that *R*_0_ can have as a function of species diversity. It is therefore constructive to provide some intuitive explanations for these types of dependencies while relating them to previous results. The explanations we now provide relate to the cases reviewed previously (cases I and II) in which we assumed, without the loss of generality, that α>1, i.e., the vector prefers host 2, and *x* represents the proportion of the non-preferred host (i.e., host 1). We begin with the assumption that γ = 1 and later we relax it and refer to the more general case where γ≠1.

When all the hosts have identical transmission ability (γ = 1) (equation 8a), the form of *R_0_*(*x*) is determined exclusively by the vector preference α (α≠1). Interestingly, our study indicates that *R_0_* is higher when γ = 1 and α≠1 than when γ = α = 1. Indeed, with the aid of equation 6 it can be verified that the inequality *R*_0γ_(x)>*R*_0αγ_(*x*) = *Vk* ^2^ *g*_1_/*Nd* is always true for 0<*x*<1, where *R*_0γ_(*x*) and *R*_0αγ_(*x*) are the values of *R*_0_(*x*) when γ = 1, α≠1 and γ = α = 1, respectively (as noted earlier, when γ = α = 1, *R*_0_  =  *Vk* ^2^ *g*_1_/*Nd* is a constant independent on *x*). This special case of diversity amplification (equation 8a) resembles previous findings of field and theoretical studies which indicated that heterogeneous biting (which occurs in our study due to vector preference, α≠1) within a single host population for which all its individuals are identical with respect to their transmission ability (γ = 1), increases *R_0_* [27], [31], [32], [37]. The diversity amplification in this case (γ = 1, equation 8a) occurs since the vector preference (α≠1) causes the number of bites the preferred group acquires to be relatively high compared to its proportion in the population. When more bites are targeted at fewer hosts, the disease transmission is enhanced. Yet, the model analysis expands on previous results by quantifying the dependency of *R_0_* on host composition (*x*), and calculating under which proportion of the non-preferred group, *x_m_*, *R*_0_ would be maximal (see equation 9, *x_m_* = α/(1+α) for γ = 1) or above the outbreak threshold.

In cases where γ≠1, equation 8b indicates that diversity amplification occurs also when the preferred host has high enough transmission ability. Under such circumstances, a relatively low proportion of the preferred host can spread the disease more efficiently than a community composed of a single species (using the same reasoning described above for γ = 1). However, when the transmission ability of the preferred host is too low (equation 7b, α>1, γ≤1/α^2^), although the number of bites therein is relatively more concentrated (as in the case of γ = 1), its low transmission ability causes *R*_0_ to decrease monotonically with an increase in its proportion in the community. The diversity amplification is therefore inhibited in this case by the insufficient transmission ability of the preferred host. For such a case the dilution affect is stronger than diversity amplification and the maximum value of *R_0_* is obtained when the community is composed of a single host with the higher transmission ability.

## Discussion

The importance of the model developed and analyzed here is that it underlines the mechanism of both, the dilution and the amplification in disease risk due to a change in the host diversity by specifying the exact conditions under which diversity amplification or dilution will occur. These conditions are formulated as a function of the vector preference and the host transmission ability. Such a general and simple formulation is able to account for the different and contradictory patterns often observed in nature (i.e., why host diversity can have opposite effects on disease risk).

For instance, Keesing et al. 2010 [10] came to the conclusion that “in recent years, a consistent picture has emerged—biodiversity loss tends to increase pathogen transmission and disease incidence.” On the other hand Loss et al (2009) [23] argued that “we found no evidence to support the hypothesis that avian richness is negatively correlated to prevalence of West Nile virus in the Chicago metropolitan area”. Another example is visceral Leishmaniasis; It was found in several studies that the proximity of humans to domestic animals is protective [38], while in other studies the disease risk increased [39]. Moreover, a recent review by Randolph and Dobson disputes the generality of the dilution effect and raises important factors that were not accounted for in previous studies which may affect the relation between host diversity and disease risk. Furthermore, they also provide alternative interpretations to existing field data [24].

### Model implications

Real world vector-borne systems usually encompass many factors which are not included in our simplified model. For instance, it can be that the total host density increases with the number of host species, thus *N* and *x* are no longer independent, or the vector density, *V*, may depend on host composition, *x*. A fixed vector population, *V*, is a reasonable assumption when the total bite rate of an individual vector is a constant independent on host species densities (equation 5, *k* = *k*_1_+*k*_2_). This is valid if the total hosts density, *N*, is high enough to support the constant bite rate as assumed by most vector borne disease models [25], [34], [36] (see also Appendix S1). A fixed host density, *N*, was assumed to delineate between host diversity (i.e., evenness and richness which are determined by host species proportions, *x*) and abundances. According to equation 6, *R*_0_ is proportional to the ratio between the vector and the total host densities, β = *V*/*N*. If, under certain biological circumstances, either the total host or/and the vector density depends on species composition, *x*, the behavior of *R*_0_(*x*) may be affected accordingly, depending on *β*(*x*) properties.

Many important field studies regarding the dilution effect have been concentrated in Lyme disease [14]–[17]. The Lyme disease vectors are ticks (*Ixodes* ssp.) [14], [16], [17] that are much less mobile than dipterian vectors such as mosquitoes and sandflies and hence are far more constrained in their host choice in field conditions. Although *Ixodes* ticks do present preference for specific host species in the lab [40], it may be that, due to their restrictive mobility, that their actual preference in the wild is much less prominent. Since the hosts of *Ixodes* ticks differ greatly in their ability to transmit the disease [16], [17], this case may be characterized by low preference (α≈1) and high transmission ratio (γ>>1), so the dilution effect that is predicated by our model (equation 7a) is consistent with the results of previous field studies on Lyme disease [14]–[17].

### Biodiversity, Global warming, and Disease risk

Considerable attention has been given to the effects that the recent decline in biodiversity [41], [42] and global warming may have on the prevalence of infectious diseases. It has been suggested that climate change (i.e., global warming) may cause some vector species to expand their range from the tropics where the host diversity is high to the temperate regions where it is low [43], [44]. In addition, biodiversity loss may also result in communities with a low number of host species [10]. Consequently, an expansion of the respective vector borne diseases may occur along the diversity gradient due to the classic dilution mechanism; i.e., a decrease in host species diversity may result in communities which are dominated by high competent hosts which will increase disease outbreaks [10], [26], [44]. Our results indicate that it is more difficult than previously thought to predict the effect of global warming and biodiversity loss on the spread of vector borne disease, since *R*_0_ is not necessarily monotonic as a function of species diversity. *R*_0_ qualitative behavior, according to our model (diversity amplification, dilution, or constant), depends on the vector preference and the transmission abilities of the hosts.

### Diversity amplification and intervention policies

Diversity amplification has implications beyond the theoretical findings described above and has the potential to affect different intervention strategies. For instance, for a population with identical individuals (γ =  1), selective use of insect repellent will cause the vectors to concentrate on the non-protected hosts which is equivalent to vector preference (α≠1). In such a case, *R_0_* may be boosted as described above (equation 8a).

In addition, in many vector borne diseases it has been reported that a relatively small proportion of the host population is responsible for a relatively high proportion of secondary infections. This is known as the 20/80 rule, i.e., 20% of the people acquire 80% of the bites [27], [45], [46]. Previous studies based on mathematical models suggested that targeted interventions which remove the entire preferred group may reduce *R_0_* by at least 80% [47].

As we have shown above, when all host individuals have equal transmission ability (γ = 1) and the vector has a preference toward a certain host subgroup, a diversity amplification occurs (equation 8a). However, since most intervention polices are able to achieve only limited reduction (i.e., less than 100%) in the proportion of the preferred group, *R_0_* may increase or decrease, depending on the amount of the reduction achieved and whether the proportion of the preferred group before the intervention was lower or higher than the proportion which maximizes *R*_0_. For example, if we plotted *R*_0_ as a function of the proportion of the preferred group (group 2) for γ = 1, the resulted graph would be hump-shaped as in figure 2. Let us therefore assume that figure 2 depicts the dependency of *R*_0_ on the proportion of the preferred group *x*. If an intervention policy reduced the proportion of the preferred group from *x*_1_≤ *x*_th2_ to *x*_2_ where *x_m_*<*x*_2_<*x*_1_, *R*_0_ would increase. However, if *x*_2_<*x_th_*_1_, *R*_0_ would decrease. Such behavior of *R*_0_ warns that under certain circumstances, an intervention policy targeted at reducing the proportion of the preferred group may have the devastating potential of increasing *R_0_* instead of reducing it. This unintuitive result challenges the common wisdom of current intervention policies [27], and calls for retrospective thinking in planning future ones.

## Conclusions

The current model exemplifies the great insight that can be gained by studying simple models [48]; although our model is much more simplistic than many natural systems, it reveals the potential complexity of the relationship between host diversity and disease risk. More specifically, the model was able to reproduce the different qualitative behaviors (diversity amplification and dilution effect) of *R*_0_ by using only two parameters; the vector preference (α), and the transmission ratio (γ). The mechanism of diversity amplification described in this study calls for retrospective thinking about the generality of the dilution effect and the relationship between infectious diseases and biodiversity. Furthermore, it was also found to be important in developing intervention policies for elimination or mitigating the morbidity of multihost vector borne diseases. This novel mechanism should therefore be regarded as an illustration of a new significant theory that merits further research.

## Supporting Information

Appendix S1 Model's supplementary analysis.
(DOC)
