# Correction: The Effects of Host Diversity on Vector-Borne Disease: The Conditions under Which Diversity Will Amplify or Dilute the Disease Risk

**DOI:** 10.1371/annotation/d3416d2f-8512-434c-a318-f69a6a6a2c60

**Published:** 2014-01-13

**Authors:** Ezer Miller, Amit Huppert

There is an error in Equation S5b, which is listed under "Appendix S1". Please view the correct Equation S5b here: http://www.plosone.org/corrections/pone.0080279.001.cn.tif

