# Supplementary material for: The Effects of Host Diversity on Vector-Borne Disease: The Conditions under Which Diversity Will Amplify or Dilute the Disease Risk
Source: PLoS One. 2013 Nov 26;8(11):e80279. doi: 10.1371/journal.pone.0080279 (PMC3841118; doi:10.1371/journal.pone.0080279)
Supplement: Appendix S1 — Model's supplementary analysis. (DOC) [file pone.0080279.s001.doc]

**Appendix S1**

Calculation of the basic reproductive rate, *R*_0_.

The basic reproduction rate of SIR type models can be calculated as the spectral radius of the so called the next generation operator [1]. Yet, to be compatible with previous multihost models, we define *R*_0_ as the square of the spectral radius [2-4]. In this definition, *R*_0_ can be classically interpreted as the number of secondary infections cased by individual sick host during the disease duration and the vector longevity. Obviously, the stability criteria, *R*_0_>1 is independent on these definitions of *R*_0_.

Calculation of the next generation operator of equation 1 yields the following 3x3 matrix **C**:

$$\mathbf{C}=\left(\begin{matrix} 0 0 \frac{k_{1}p_{1}}{d} \\ 0 0 \frac{k_{2}p_{2}}{d} \\ \frac{k_{1}q_{1}V}{\delta_{1}N_{1}} \frac{k_{2}q_{2}V}{\delta_{2}N_{2}} 0 \end{matrix}\right) (S1)$$

Where *V*, the vector population size, is assumed constant (as we noted in the main text, the vector population is at dynamic equilibrium, i.e., *r* = *d*, see also table 1). The squared magnitude of the spectral radius of operator **C** in equation S1 is the maximal squared norm of its eigenvalues. Since the matrix **C** has two real eigenvalues with identical norms, *R_0_* is:

$$R_{0}=V(\frac{k_{1}^{2}p_{1}q_{1}}{N_{1}\delta_{1}d}+\frac{k_{2}^{2}p_{2}q_{2}}{N_{2}\delta_{2}d}) (S2)$$

Calculation of the vector bite rate, *k*_i_.

In the current study we adopt the classic Holling type II functional response to represent the vector bite rate (no. of bites per unit time) [5-7]. The type II functional response is a well known model which has been verified experimentally for various animal taxa, especially predatory insects [8]. In Holling model, the number of bites an individual vector has with host species *i* per unit time, *k_i_*, is given by:

$$k_{i}=\frac{a_{i}N_{i}}{1+a_{1}h_{1}N_{1}+a_{2}h_{2}N_{2}} (S3)$$

Where *a_i_*, *h_i_*, and *N_i_* are the vector searching efficiencies, the vector handling times, and the density of host species *i* (*i* can be 1 or 2), respectively.

As for most cases of vector-borne diseases [3], we assume that the host densities are high enough so that they do not limit the vector bite rate, and the handling times of both host species are identical, i.e., *a_i_h_i_N_i_*>>1 for some *i*'s (at least one species) and *h_1_*=*h_2_*=*h*. Incorporating these assumptions into equation S3 yields,

$$k_{i}=\frac{ka_{i}N_{i}}{a_{1}N{}_{1}+a_{2}N_{2}} (S4)$$

Where *k* is the total bite rate of the vector with the entire hosts community, i.e., *k*=*k_1_*+*k_2_*=*1/h*. If we now make use of the expressions for the vector preference (α) and the transmission ratio (γ) given in equations 3 and 4, and using *x*, the proportion of species 1 in the community, we have:

$\begin{matrix} k_{1}=\frac{ka_{1}N_{1}}{a_{1}N_{1}+a_{2}N_{2}}=\frac{kNx}{Nx+\alpha N(1-x)}=\frac{kx}{x+\alpha(1-x)} (S5a) \\ k_{2}=\frac{ka_{2}N_{2}}{a_{1}N_{1}+a_{2}N_{2}}=\frac{k\alpha N(1-x)}{Nx+\alpha N(1-x)}=\frac{k\alpha x}{x+\alpha(1-x)} (S5b) \end{matrix}$

The approximations of equation S5 has been used before to represent the vector bite rate with respect to different host species but without relating it to Holling models of functional response [9-11]. It is interesting to note that equation S5 were also obtained from the ideal free distribution theory, were host individuals are assumed as resource patches that are utilized by the vector [12]. Equation S5 forms an extension of density-independent (frequency-dependent) contact rate (*k* is independent on *N*_1_ and *N_2_*) to a two-host vector borne disease.

Finding the conditions under which *R*_0_ is monotonic (dilution effect) or

hump-shaped (diversity amplification), as a function of species

composition, *x*.

Analysis of *R*_0_(*x*) given in equation 6 indicates that if:

$$0<x_{m}=\frac{\alpha}{1-\alpha}+\frac{2\alpha^{2}\gamma}{\alpha^{2}\gamma-1}<1 (S6)$$

*R*_0_ is hump-shaped as a function of *x*, and when:

$$\frac{\alpha}{1-\alpha}+\frac{2\alpha^{2}\gamma}{\alpha^{2}\gamma-1}\geq1 or \frac{\alpha}{1-\alpha}+\frac{2\alpha^{2}\gamma}{\alpha^{2}\gamma-1}\leq0 or \frac{\alpha}{1-\alpha}+\frac{2\alpha^{2}\gamma}{\alpha^{2}\gamma-1}\in\varphi(S7)$$

*R*_0_ is monotonic increasing with the proportion of the species with the highest transmission ability, provided that either α≠1 or γ≠1. By solving the above inequalities (equations S6 and S7), it can be shown that the conditions for diversity amplification (hump-shaped *R*_0_) are:

And the conditions for dilution (monotonic *R*_0_) are:

The conditions in equations S8 and S9 are sufficient and necessity, yet, they are rather complicated. It is therefore constructive to look at narrower, sufficient conditions which are more easily understood. From equations S8 and S9, sufficient conditions for a monotonic *R_0_*(*x*) are:

α>1 & γ<1/α^2^ (S10a)

α=1 & γ≠1 (S10b)

and sufficient conditions for a hump-shaped *R_0_*(*x*) are:

α≥2 & γ>1 (S11a)

α≠1 & γ=1 (S11b)

**References**

1. van den Driessche P, Watmough J (2002) Reproduction numbers and sub-

threshold endemic equilibria for compartmental models of disease transmission. Math Biosci 180: 29-48.

1. Rogers DJ (1988) The dynamics of vector –transmitted diseases in human

communities. Philos T R Soc B 321: 513-539.

1. Ross R (1910) The prevention of malaria. London: John Murray. 669 p.
2. Dye C, Hasibeder G (1986) Population dynamics of mosquito-borne disease –

effects of flies which bites some people more frequently than others.

T Roy Soc Trop Med H 80: 69-77.

5. Holling CS (1959) Some characteristics of simple types of predation and

parasitism. Can Entomol 91: 385-398.

6. Holling CS (1966) The functional response of invertebrate predators to prey

density. Mem Entomol Soc Can 48: 1-86.

7. Antonovics J, Iwasa Y, Hassell MP (1995) A generalized model of parasitoid,

venereal, and vector-based transmission processes. Am Nat 145:

661-675.

8. Hassel PM (1978) The dynamics of Arthropod predator-prey systems. 1 ed. New

Jersey, Princeton University Press.

9. Sota T, Mogi M (1989) Effectiveness of zooprophylaxis in malaria control-a

theoretical inquiry with a model for mosquito populations with 2 bloodmeal

hosts. Med Vet Entomol 3: 337-345.

1. Simpson JE, Hurtado PJ, Medlock J, Molaei G, Andreadis TG, et al. (2012)

Vector host-feeding preferences drive transmission of multi-host pathogens:

West Nile Virus as a model system. Proc Roy Soc B-Biol Sci 279: 925-933. 11. Yakob L, Bonsall MB, Yan G (2010) Modeling *Knowlesi* malaria transmission

In humans: vector preference and host competence. Malaria J. 9.

12. Kelly DW, Thompson CE (2000) Epidemiology and optimal foraging: modeling

the ideal free distribution of insect vectors. Parasitology 120: 319-327.
